# Broccoli Sprouts Promote Sex-Dependent Cardiometabolic Health and Longevity in Long-Evans Rats

**DOI:** 10.3390/ijerph192013468

**Published:** 2022-10-18

**Authors:** Ronan M. N. Noble, Forough Jahandideh, Edward A. Armstrong, Stephane L. Bourque, Jerome Y. Yager

**Affiliations:** 1 Department of Pediatrics, University of Alberta, Edmonton, AB T6G 2G3, Canada; 2 Women and Children’s Health Research Institute, University of Alberta, Edmonton, AB T6G 1C9, Canada; 3 Department of Anesthesiology & Pain Medicine, University of Alberta, Edmonton, AB T6G 2G3, Canada; 4 Department of Pharmacology, University of Alberta, Edmonton, AB T6G 2G3, Canada

**Keywords:** aging, behavior, blood pressure, broccoli sprouts, cardiovascular function, glucose metabolism, longevity

## Abstract

Antioxidants and anti-inflammatory compounds are potential candidates to prevent age-related chronic diseases. Broccoli sprouts (BrSp) are a rich source of sulforaphane—a bioactive metabolite known for its antioxidant and anti-inflammatory properties. We tested the effect of chronic BrSp feeding on age-related decline in cardiometabolic health and lifespan in rats. Male and female Long-Evans rats were fed a control diet with or without dried BrSp (300 mg/kg body weight, 3 times per week) from 4 months of age until death. Body weight, body composition, blood pressure, heart function, and glucose and insulin tolerance were measured at 10, 16, 20, and 22 months of age. Behavioral traits were also examined at 18 months of age. BrSp feeding prolonged life span in females, whereas in males the positive effects on longevity were more pronounced in a subgroup of males (last 25% of survivors). Despite having modest effects on behavior, BrSp profoundly affected cardiometabolic parameters in a sex-dependent manner. BrSp-fed females had a lower body weight and visceral adiposity while BrSp-fed males exhibited improved glucose tolerance and reduced blood pressure when compared to their control counterparts. These findings highlight the sex-dependent benefits of BrSp on improving longevity and delaying cardiometabolic decline associated with aging in rats.

## 1. Introduction

Aging is a complex biologic process associated with structural and biochemical changes that result in progressive functional decline [1]. Free radical and related oxidants are believed to be involved in this process. Oxidative stress is thought to activate the immune system generating inflammatory responses that further exacerbate oxidative stress; the resulting cyclic pattern leads to progressive physiological decline, culminating into increased morbidity, and eventual mortality [2]. Uncontrolled oxidative stress has been suggested as one of the primary causes of cardiovascular disease and other major health problems [3]. Reduction of oxidative stress on the other hand, has the potential to reverse the age-related functional deficits in rodents [4,5]. Antioxidant molecules, acting directly or indirectly to protect cells against oxidative stress, have the potential to prevent age-related diseases. Direct antioxidants, such as ascorbate and tocopherols (exogenous antioxidants) as well as glutathione, α-lipoic acid, and ubiquinols (endogenous antioxidants), are redox active compounds able to scavenge reactive oxygen species. Direct antioxidants are typically short-lived, requiring replenishment or regeneration as they are consumed in mediating their antioxidant effects. In contrast, indirect antioxidants mediate their effects by supporting endogenous antioxidant function and are more efficient in reducing oxidative stress and its associated dysfunctions. Indirect antioxidants are able to activate the Kelch-like ECH-Associating protein 1 nuclear factor erythroid 2 related factor 2-antioxidant response element (Keap1-Nrf2-ARE) pathway, an important cellular defense mechanism. Keap1-Nrf2-ARE regulatory pathway activation results in transcriptional induction of various cytoprotective proteins that play a role in the regeneration of the direct antioxidant glutathione [6]. Age-associated decline in Nrf2 signaling activity has been suggested as an important factor in the development of age-related onset of neurodegenerative disorders [1]. Restoration of endogenous cytoprotective mechanisms through increased Nrf2 activity is believed to be an effective strategy to protect against age-related physiological decline [1].

Many functional foods containing bioactive compounds with antioxidant and anti-inflammatory properties are promising agents for the prevention of age-related chronic diseases [7]. Sulforaphane is an aliphatic isothiocyanate and an Nrf2 activator with antioxidant properties [8]. Broccoli sprouts (BrSp) contain the highest concentration of glucoraphanin, a glucosinolate hydrolyzed by myrosinase to sulforaphane. Crushing of the plant cells by chewing or food preparation releases myrosinase from intracellular vesicles, after which glucoraphanin hydrolyze to yield sulforaphane. Young BrSp contain higher amounts of glucosinolates (20–50 times more) than mature market-stage broccoli [9] and may therefore be a better choice for its health-promoting properties. Indeed, we have shown that maternal BrSp feeding during pregnancy confers protection and mitigates brain injury and neurodevelopmental delays in a rat model of induced intra-uterine growth restriction and prenatal inflammation [10,11]. In adult rodents, long-term consumption of BrSp promotes cardiovascular and metabolic health and mitigates organ damage in diabetes and hypertension [12,13,14]. Despite these well documented effects, the long-term effects of BrSp feeding on age-related physiological decline has yet to be reported. Therefore, the aim of this study was to determine the effects of long-term BrSp feeding on cardiometabolic health, behavioral traits, and longevity in rats. Since age-related complications have shown a sex-specific pattern [15], we studied both male and female rats.

## 2. Materials and Methods

### 2.1. Animals and Treatments

The experimental protocols described herein were approved by the University of Alberta Animal Care and Use Committee in accordance with the guidelines established by the Canadian Council of Animal Care. Twelve-week-old Long-Evans rats (bred in-house) and housed at the University of Alberta animal care facility under a light/dark cycle of 12 h and an ambient temperature of 22 °C with 40–60% relative humidity. Fifty-six rats were bred in total. An estimated standard deviation of 20% of the mean, an overall effect of 25% for cardiometabolic parameters with 80% power and an α of 0.05 meant a sample size of at least 10 per group was required. All rats had *ad libitum* access to a standard rodent chow (5L0D; PicoLab, St Louis, MO, USA) and water throughout the study. At 12 weeks of age, males and females were randomized to the control or BrSp groups. Body weight was monitored monthly for all animals starting at 4 months of age. 

Broccoli seeds were purchased from Mumm’s Sprouting Seeds (Parkside, SK). Seeds were sprouted for 4 days and greened for 4 h before harvesting. The sprouts were then air dried for 7 days before use. Rats were fed 300 mg/kg body weight BrSp 3 days per week (Monday, Wednesday, Friday, to limit rat agitation) beginning at 4 months of age until death/euthanasia. A quantity of 300 mg/kg BrSp was the maximum amount Long-Evans rats ate entirely in our pilot experiments and is consistent with amounts used in other studies [14,16].

### 2.2. Body Composition and Metabolic Parameters

Body composition as well as oral glucose tolerance tests (oGTT) and insulin tolerance tests (ITT) were assessed in all rats after 0, 6, 12, 16, and 18 months of treatment (corresponding to 4, 10, 16, 20, and 22 months of age, respectively). Body composition was assessed in conscious rats using a whole-body composition analyzer (EchoMRI 4-in-1/1000, Echo Medical Systems LLC, Houston, TX, USA) throughout the study. For oGTT, rats were fasted overnight (16 h). Baseline blood glucose levels were assessed using a glucometer (AccuChek Aviva Nano, Roche Diagnostics) from a saphenous venipuncture. Rats were then administered a glucose solution (0.5 g D-glucose per mL water) by oral gavage at a dose of 2 g/kg body weight. Blood glucose concentrations were assessed at 15, 30, 60, 90, and 120 min post-glucose administration by saphenous venipuncture. 

For the ITT, rats underwent a fast of 4 h prior to blood sampling. Baseline glucose levels were assessed from a saphenous venipuncture using an AccuChek Aviva Nano blood glucometer (Roche Diagnostics). Rats then received an intraperitoneal injection of insulin (1.0 IU/kg body weight for males or 0.75 IU/kg body weight for females). Blood glucose concentrations were then assessed at 15, 30, 60, 90, and 120 min post-insulin injection by saphenous venipuncture.

### 2.3. Tail-Cuff Plethysmography 

Systolic blood pressure was measured non-invasively by tail-cuff plethysmography (CODA, Kent Scientific, Torington, CT, USA). Animals were lightly sedated (1% isoflurane, 1 L/min oxygen via nose-cone) and maintained on a temperature-controlled platform (kept at 37 °C). After equilibrating to isoflurane for 10 min, a total of 20 separate BP recordings (cycles) were taken in a single session. Integrity of BP measurements were evaluated based on whether meaningful diastolic BP and heart rate recordings could be collected in tandem, though these parameters were not analyzed. Systolic BP data from any cycle where heart rate or diastolic BP recordings could not be calculated were excluded from analysis.

### 2.4. Echocardiography 

Rats were lightly anaesthetized (2.0% Isoflurane, 1 L/min oxygen via nose-cone), kept on a temperature-controlled warming pad (kept at 37 °C), and imaged in the supine position using a high-resolution imaging system and a 16–23 Mhz transducer (Vevo2100, VisualSonics Inc., Toronto, ON, Canada). A single operator who was blinded to experimental groups performed all assessments. Parasternal short axis M mode tracings of the left ventricle (LV) were recorded at the widest point of the heart, with the M mode cursor placed perpendicular to the anterior and posterior wall of the left ventricle. LV end-diastolic and end-systolic diameters (LVID), and septal (IVS) and posterior wall (LVPW) thicknesses were measured. LV mass was calculated according to the troy formula (LV mass = 1.053 × 0.8 × ([LVIDd + LVPWd + IVSd]^3^ − [LVIDd]^3^)), where 1.053 is the specific gravity of the myocardium. Stroke volume (SV) and cardiac output (CO) were calculated with ventricular end-systolic and end-diastolic volumes obtained from M mode images. The trans mitral flow velocity was obtained from the apical four chambers view. The ratio of peak early filling (E) and atrial velocity (A) was measured with pulse wave Doppler imaging. E and A wave images were used to quantify isovolumic relaxation and contraction time, as well as aortic ejection time, which were then combined to calculate a TEI index. All values were measured under steady state conditions and averaged from three cardiac cycles taking care to exclude cycles that took place during inhalation.

### 2.5. Behavioral Tests 

All behavioral tests were performed after 14 months of treatment (i.e., at 18 months of age). For the tapered beam, hind limb function was determined using the raised tapered beam test [17] Tapered beam test provides a sensitive measure of bilateral motor function based on foot faults (slips) made by a rodent traversing a gradually narrowing beam [18]. Briefly, animals walked the beam in two consecutive recorded trials, albeit only the second trial was scored. Half faults (score = 1) were defined as an instance in which a portion of the hind paw used the side or lower ledge for weight bearing. Full foot faults (score = 2) were recorded when the full hind paw used the bottom ledge for support. Performance in the tapered beam task was assessed by analyzing the sum of the foot faults during the second trial. 

The elevated plus maze was used to assess anxiety-related behavior in rats, as well as measures of locomotion [19]. Rats were placed in an elevated structure with two opposing enclosed arms (15 cm high walls) and two opposing open arms. Each arm was 10 cm wide and 50 cm long. Rats were placed in the center of the apparatus, facing an open arm and behavior was recorded for 5 min. Recordings were analyzed for entries and time spent in each arm type (open versus closed).

Finally, the open field test was used to assess exploratory activity in an unfamiliar environment; this test also provides measures of anxiety-related behaviors [20], as well as locomotor activity. Rats were placed in the center of an opaque box (60 cm × 60 cm × 60 cm) and video recorded for 5 min to track exploratory and thigmotactic behaviors [21]. Time spent in the center region of the box (defined as an average body length from the edge of the box) and total distance travelled were recorded and analyzed using tracking software. 

### 2.6. Lifespan Estimation 

Animals were inspected twice weekly for general health status. Established criteria for humane endpoints were used to estimate lifespan, and evaluations were performed by a clinical veterinarian. Criteria for humane endpoints included excessive loss of body weight (>20% of body weight from their top weight), breathing abnormalities (respiratory distress, labored breathing, increased or decreased respiratory rate, cyanosis), uncontrolled bleeding, presence of big (>3 cm) or ulcerated and necrotic tumors, limb paralysis, dermatitis, malocclusion, or behavioral indicators of pain or severe distress (e.g., hunched posture, extensive porphyrin staining, piloerection). Post-mortems were performed on euthanized rats, as well as those found dead in their cages.

### 2.7. Statistical Analyses

Data are presented as mean ± SEM. Outcomes were analyzed by two-way ANOVA for the effects of intervention (BrSp-feeding) and time, followed with Holm–Sidak post hoc test where main effects were found. Area under the curve (AUC) and behavioral data were analyzed either by Student’s t-test or Mann–Whitney test as appropriate. Data were assessed for normality by the Shapiro–Wilk test prior to analyses. All statistical analyses were conducted using Prism 9.0 (GraphPad Software Inc., San Diego, CA, USA).

## 3. Results

### 3.1. Survival and Mean Age at Death

**Figure 1:**
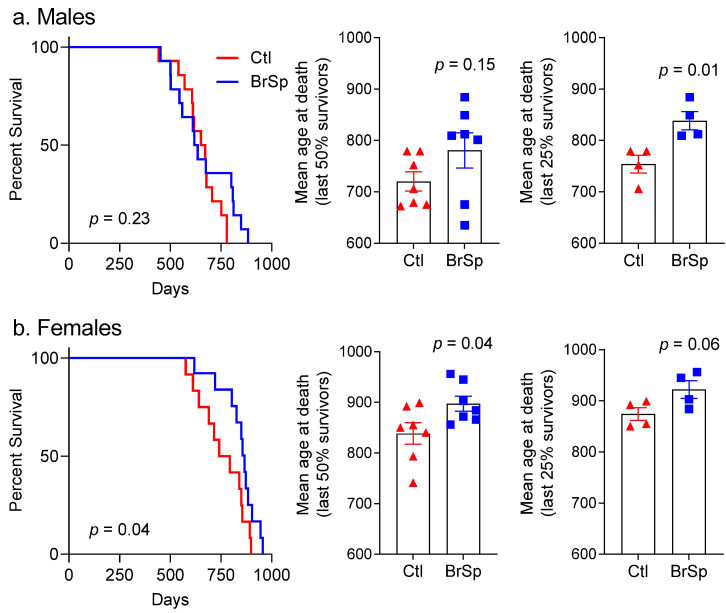
Survival in (**a**) male and (**b**) female rats supplemented with or without broccoli sprouts (BrSp). Kaplan–Meier curves showing survival in (**a**) males and (**b**) females. Middle panels depict mean age at death of the oldest 50% survivors of male and female rats. Similarly, right panels depict mean age at death of the oldest 25% survivors of male and female rats.

Offspring lifespan, estimated based on established criteria for humane endpoints, are shown in Figure 1. Average age at the time of death in male rats was 648 ± 25 days (Ctl), and 661 ± 39 (BrSp), and in female rats was 726 ± 41 (Ctl) and 826 ± 29 (BrSp). Log-rank analysis revealed no overall effect of BrSP-feeding on lifespan in males (*p* = 0.26, Figure 1a, left). However, survival curves of control and BrSp-fed male rats tended to separate at older ages (beyond the point of 2 years), and while there were no differences in lifespan in the oldest 50% of surviving rats (Figure 1a, middle), mean age at death for the oldest 25% of rats was higher in BrSp-fed rats (838 ± 18 days) than controls (754 ± 17 days; *p* = 0.01, Figure 1a, right). In females, BrSp feeding improved survival, and the benefits were evident when considering all rats (*p* = 0.04, Figure 1b, left), as well as the oldest 50% of survivors (Figure 1b, middle), although differences in the oldest 25% of female survivors did not reach statistical significance (*p* = 0.06; Figure 1b, right). 

### 3.2. Cause of Death

With the exception of 4 males (1 Ctl and 3 BrSp-fed) and 1 female (1 Ctl) found deceased, all rats were euthanized based on criteria for humane endpoints. The criteria used to determine humane endpoints are summarized in Table S1. Weight loss and presence of tumors were the predominant criteria for euthanasia. Cases of malocclusion and severe dermatitis were also considered causes for euthanasia; however, since these conditions would otherwise not be considered terminal, these subjects were censured, and therefore not included in survival calculations show in Figure 1. Co-morbidities evident at postmortem are also shown in Table S2. Evidence of cardiopulmonary disease and complications of the kidney, liver, and brain function were present in all animals at the time of death, though it is unclear whether any or all of these complications contributed to morbidity and mortality. Table 1 summarizes the data on the number of rats with more than one morbidity (cardiopulmonary, kidney, liver, brain, or tumor) at the time of death.

### 3.3. Body Weight and Composition

**Figure 2:**
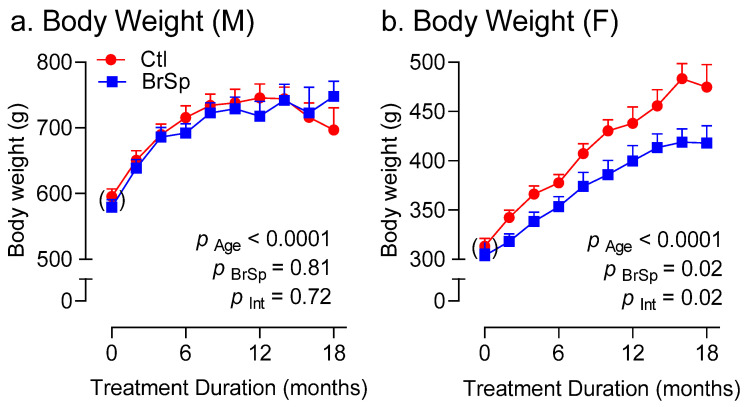
Body weight over time in (**a**) male and (**b**) female rats. Data is presented as Mean ± SEM for *n* = 6–14 offspring from separate litters in each group and analyzed by two-way ANOVA.

**Figure 3:**
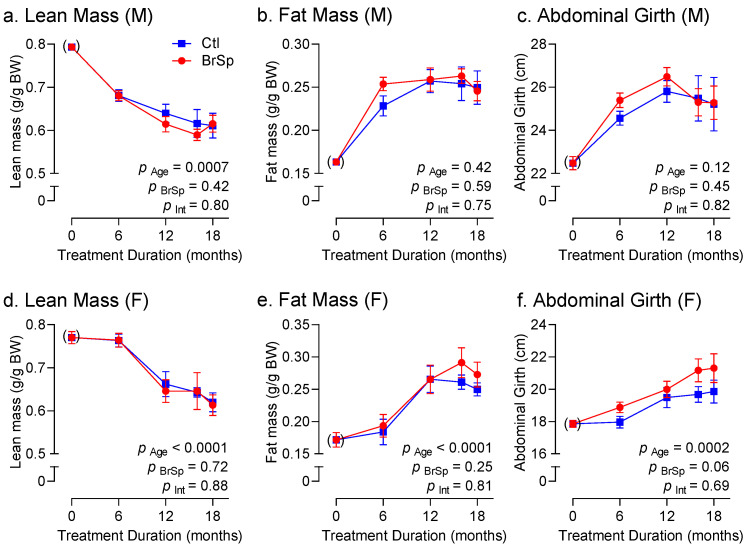
Body composition and waist circumference measures in (**a**–**c**) males and (**d**–**f**) females, including (**a**,**d**) lean mass, (**b**,**e**) fat mass, and (**c**,**f**) abdominal girth. Data is presented as Mean ± SEM for *n* = 6–14 rats in each group and analyzed by two-way ANOVA.

Body weight in male offspring was not affected by BrSp feeding (Figure 2a). In contrast, BrSp-fed females gained less weight compared to their control counterparts (Figure 2b). As expected, aging was associated with changes in body composition (Figure 3). All rats, irrespective of sex or treatment, tended to gain fat (Figure 3a,d) at the expense of lean mass (Figure 3b,e) as they aged. BrSp had no effect on age-related changes in body composition in either sex. Interestingly, whereas abdominal circumferences, a surrogate of abdominal adiposity, was unaffected by BrSp-feeding in males (P _BrSp_ = 0.35, Figure 3c), BrSp-feeding tended to mitigate this increase in females compared to their control counterparts (P _BrSp_ = 0.06, Figure 3f). 

### 3.4. Glucose Homeostasis

**Figure 4:**
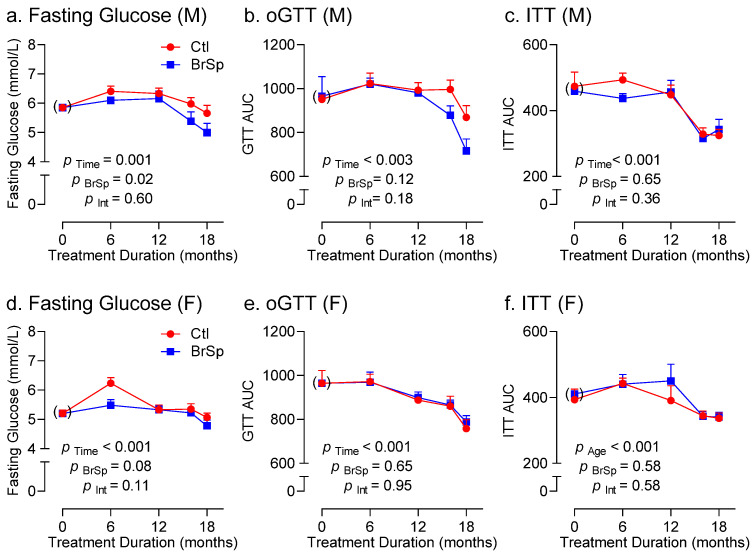
Changes in (**a**,**d**) fasting blood glucose and area under the curve for (**b**,**e**) GTT, and (**c**,**f**) ITT in (**a**–**c**) male and (**d**–**f**) female rats over time. Data is presented as Mean ± SEM for *n* = 6–14 offspring from separate litters in each group and analyzed by two-way ANOVA.

BrSp modestly affected glucose handling parameters, albeit in a sex-dependent manner (Figure 4). BrSp-feeding was associated with reduced fasting blood glucose levels in males (P_BrSp_ = 0.02, Figure 4a), although no differences were apparent in glucose (Figure 4b) or insulin sensitivity (Figure 4c). In contrast, BrSp-feeding did not improve fasting glucose levels (Figure 4d), nor glucose or insulin sensitivity (Figure 4d) in females.

### 3.5. Blood Pressure and Cardiac Function

**Figure 5:**
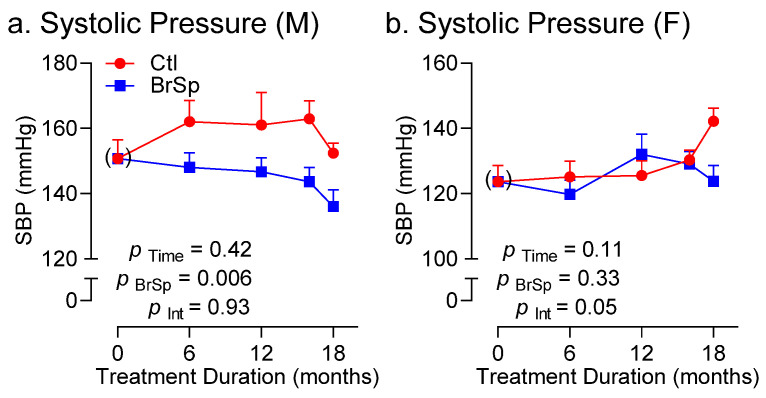
Systolic blood pressure in (**a**) male and (**b**) female offspring over time. Data is presented as Mean ± SEM for *n* = 6–14 offspring from separate litters in each group and analyzed by two-way ANOVA.

BrSp-feeding had a pronounced blood pressure lowering effect in males that was evident in all ages (Figure 5a). Consistent with these data, echocardiographic measurements revealed reduced cardiac size in BrSp-fed males compared to controls, reflected in left-ventricular internal diameter at end-diastole (LVIDd), as well as in corrected left ventricular mass (Table 2). Cardiac output was also reduced in BrSp-fed males compared to controls as indicated by a significant interaction between time and treatment (P _Int_ = 0.004, Table 3), whereas E/A ratio tended to increase upon BrSp feeding in males (P _Int_ = 0.06, Table 3).

BrSp-feeding was also associated with a blood pressure lowering effect in females, albeit this was largely attributed to differences recorded after 18 months of treatment (Figure 5b). This effect could not be attributed to attrition, since paired analysis of those female rats surviving at 16 and 18 months of age yielded similar systolic blood pressure differences (Ctl-F (16 mo.): 132 ± 3 mmHg [*n* = 9]; BrSp-F (16 mo.): 128 ± 4 mmHg [*n* = 11]; *p* = 0.57; Ctl-F (18 mo.): 142 ± 4 mmHg [*n* = 9]; BrSp-F (18 mo.): 124 ± 5 mmHg [*n* = 11]; *p* = 0.01). BrSp-feeding had modest effects on cardiac morphology and function in the females, albeit an interaction was observed in corrected LV mass, attributed to an un-sustained increase in controls at the penultimate time point (16 months; Table 2). Finally, like males, cardiac output also tended to be lower in BrSp-fed females (Table 3).

### 3.6. Behavioral Analysis

Table 4 summarizes the parameters measured for activity and anxiety behavioral traits in rats. Male and female rats in both treatment groups performed similarly in the open field maze when total time in center and total distance was measured. In contrast, total mobile time was higher in BrSp-fed males (*p* = 0.03) but not females (*p* = 0.13). No differences were observed in performance in the elevated plus maze parameters between the control and BrSp groups in either sex. On the tapered beam test, BrSp-fed females tended to have fewer left foot faults compared to their control counterparts (*p* = 0.06), whereas no such differences were evident between treatment groups in males. 

## 4. Discussion

Aging is characterized by a decline of cellular, tissue, and organ function, and is typically associated with a loss of homeostasis and decreased adaptability to stress. This loss of functionality and diminished adaptability yields a greater vulnerability to disease and mortality [22]. Indeed, aging is the main risk factor for cardiovascular and metabolic diseases, which have become particularly pressing issues as the global aging population continues to grow. Functional foods contain bioactive compounds that, in many cases, promote health due to their pleiotropic effects, and thus may mitigate age-related functional decline and improve longevity. The objective of this study was to assess the effects of long-term BrSp feeding on longevity in rats, as well as on cardiometabolic health parameters. To summarize the outcomes, BrSp feeding of rodents, starting at 4 months of age caused (1) extended life span in rats, albeit this was observed predominantly in females; (2) reduced body weight gain in females; (3) modest improvements in glucose handling in males; (4) marked blood pressure reduction in males; and (5) modest changes in behavioral traits in both sexes. 

As expected, aging was associated with an increase in body weight in all rats, which was accompanied by a decrease in lean mass and concomitant increase in fat mass. The increase in abdominal girth, which has been reported to be a reliable surrogate of abdominal fat [23], paralleled the increase fat mass, suggesting the age-related increase in fat is largely attributed to accumulation of visceral fat. However, in BrSp-fed females, rise in abdominal girth tended to be lower, which may indicate a propensity for more subcutaneous, and hence healthier, fat accumulation compared to control females. Yet despite the tendency to reduced abdominal fat accumulation, there were no apparent changes in glucose handling (i.e., fasting glucose or glucose tolerance) in females. In contrast, only male BrSp-fed rats exhibited improved fasting glucose levels despite no differences in body composition, which is consistent with other reports in male rats using sulforaphane [1], and underscores that body composition is by no means the sole determinant of glucose homeostasis. This is perhaps best exemplified by the observation that among all groups over time, fasting glucose levels as well as GTT and ITT AUCs remained largely unchanged (or even decreased), despite increases in body weight and fat deposition, which has been reported by others [24,25,26].

The pronounced sexually dimorphic effect of BrSp-feeding, aside from body weight gain observed only in females, was on BP regulation in males. At all times assessed, BrSp feeding caused a 10–15 mmHg reduction in systolic BP, which coincided with lower LV mass (assessed by echocardiography), reflecting a diminished cardiac afterload [27]. The benefits of blood pressure reduction on longevity are well documented [28], and studies indicate that even modest reductions of 1–2 mmHg can impart survival benefits [29]. Although overall survival in males was not affected by BrSp-feeding, the oldest surviving males were invariably BrSp-fed. In contrast, BP profiles of control and BrSp-fed females were largely superimposable throughout the experiments, and yet the latter group exhibited a clear survival advantage. The survival benefit could be attributed, in part, to BrSp-feeding mitigating the marked rise in BP in control rats at 24 months of age (after 18 months of treatment)—a consistent finding among all control rats assessed at that time (*n* = 9), suggesting this was not a spurious observation. Although the cause of this BP rise is unclear, it may be attributed, in part, to hormonal changes. The increasing incidence of hypertension occurring at older ages in women than in men [30], is attributed to hormonal changes following menopause; hormonal changes accompanying reproductive senescence in rats may occur at even more advanced ages [31,32]. Therefore, it is not altogether unexpected that marked BP rises occur beyond 18 months of age. Other potential mechanistic effects may be attributed to the vascular inflammation seen in spontaneously hypertensive rats. Wu et al. [33], found that feeding BrSp to spontaneously hypertensive rats reduced the presence of intra-luminal inflammatory cells seen on pathologic examination, and this coincided with a reduction in the onset of hypertension. However, the latter does not explain the sex differences seen in our study, as the former did not compare the sexes. 

Notwithstanding, how BrSp mitigates this late-stage BP rise remains unclear. Despite the overall modest effects of BrSp on the measured cardiometabolic parameters in females, survival benefit was quite compelling in all the females; BrSp-fed females lived longer than their control counterparts. The small albeit significant reduction in cardiac output could indicate an overall decrease in metabolic demands in females, which has been linked to longevity [34,35]. However, cardiac output was sporadically reduced in BrSp-fed males as well, which may explain the less robust survival advantage in this group. 

Aging is associated with structural and biochemical changes that culminate in profound functional changes over time; increased tendencies to anxiety and depression-like behaviors [36,37,38] and impaired performance in tasks requiring coordinated control of motor and reflex responses [39] have been described in aged rodents. To this end, control and BrSp-fed rats underwent a series of behavioral tests, including the tapered beam test, which evaluates motor coordination and balance, as well as elevated plus maze and open field tests, which evaluate locomotor activities as well as anxiety-like behaviors, respectively [20]. BrSp-fed females tended to have fewer foot faults than their control counterparts, which could reflect improved neurological function with treatment in these animals. In contrast, no apparent improvements in locomotor function were evident in males. However, BrSp-fed males exhibited increased time active within the open field, which may reflect better exploratory activity compared to control counterparts. Despite this benefit, though other well-documented outcomes reflecting anxiety (e.g., time spent in the center of the open field and ventures into the open-arms of the elevated plus maze) were not affected by BrSp in males or females. Notably, the outcomes of these tests probe state-like anxiety, in which situations create anxiety [40], rather than trait anxiety, which reflect fears that are not apparent to others [41] Therefore, it is possible that the beneficial effects of BrSp on trait-anxiety may be more pronounced, and thus future studies investigating whether this treatment may be useful in mitigating anxiety-like behaviors in other contexts requires further investigation.

There are other limitations to this study that warrant discussion. First, because the primary objective of this study was to assess longevity, post-mortem tissue collection invariably occurred from previously moribund animals, thus precluding biochemical analyses. As such, measures of oxidative stress and inflammation (such as reactive oxygen species and malondialdehyde) were not measured in this study. Therefore, mechanisms by which long-term BrSp-consumption promotes health and longevity remain unclear. However, the present study sets the stage for such mechanistic studies, which in turn may provide insights into the sex-differences in beneficial effects of BrSp reported herein. Similarly, variability from time of death to post-mortem tissue collection introduces a large error in histological and immunohistochemical examination. It is well known that small sample sizes decrease statistical power and decrease the flexibility of effect sizes. There is the potential that this study was underpowered and larger groups may have resulted in significance (e.g., Figure 1). Finally, despite low calorie content of BrSp, it is possible that BrSp feeding may have affected overall food consumption. Although intermittent food intake assessments did not reveal differences between control and BrSp-fed rats in either sex (data not shown), the use of non-purified rodent chow, which are less consistent in their nutritional make-up and indeed less nutritive overall than purified diets, made food intake parameter difficult to assess. Whether BrSp affects satiety is important because the effects of reduced food consumption on longevity are well documented

## 5. Conclusions

Overall, this study clearly demonstrated the positive effects of chronic BrSp consumption on longevity in rats. Body weight, visceral adiposity, glucose handling, and blood pressure were positively affected by BrSp consumption in rats throughout their life span in a sex-specific manner. Rats fed with BrSp also exhibited improved activity and bilateral motor function when older. This highlights the potential of chronic BrSp consumption on improving cardiometabolic health and neurological dysfunctions associated with aging, which can eventually lead to improved longevity in the aging population. 

## Figures and Tables

**Table 1:** Number and % of rats with more than one health complication at the time of death *.

Groups	N	Total	%
Male-Ctl	11	13	84.6%
Male-BrSp	8	12	66.7%
Female-Ctl	8	11	72.7%
Female-BrSp	7	12	58.3%

* Rats found dead (*n* = 5) or euthanized prematurely solely due to malocclusion (*n* = 3) are excluded.

**Table 2:** Echocardiographic measurements (cardiac morphometry) in rats fed a control or BrSp diet.

		Ctl					BrSp					*p-*Value		
		0 Months	6 Months	12 Months	16 Months	18 Months	0 Months	6 Months	12 Months	16 Months	18 Months	Time	BrSp	Int
Male Offspring	IVSd, mm	1.7 ± 0.0	2.2 ± 0.1	2.4 ± 0.1	2.2 ± 0.1	2.4 ± 0.1	1.7 ± 0.0	2.2 ± 0.1	2.4 ± 0.1	2.1 ± 0.0	2.2 ± 0.1	**0.008**	0.37	0.51
	IVSs, mm	2.3 ± 0.3	3.6 ± 0.2	3.8 ± 0.2	3.6 ± 0.1	4.0 ± 0.2	2.3 ± 0.3	3.6 ± 0.1	4.0 ± 0.1	3.5 ± 0.1	3.8 ± 0.2	**0.02**	0.85	0.40
	LVIDd, mm	7.9 ± 0.6	8.4 ± 0.2	8.8 ± 0.2	9.2 ± 0.3	8.8 ± 0.2	8.0 ± 0.3	8.3 ± 0.2	8.1 ± 0.2	8.2 ± 0.5	8.4 ± 0.4	0.61	**0.04**	0.31
	LVIDs, mm	3.7 ± 0.6	4.2 ± 0.3	4.4 ± 0.3	4.5 ± 0.3	4.0 ± 0.4	3.7 ± 0.6	3.8 ± 0.3	3.5 ± 0.1	3.9 ± 0.4	3.7 ± 0.3	0.75	0.18	0.60
	LVPWd, mm	2.2 ± 0.2	2.2 ± 0.1	2.3 ± 0.1	2.0 ± 0.2	2.3 ± 0.2	2.2 ± 0.2	2.3 ± 0.1	2.1 ± 0.1	2.1 ± 0.2	2.2 ± 0.1	0.72	0.88	0.78
	LVPWs, mm	3.4 ± 0.1	3.6 ± 0.2	3.7 ± 0.1	3.7 ± 0.2	3.8 ± 0.2	3.5 ± 0.0	3.8 ± 0.1	3.6 ± 0.1	3.6 ± 0.1	3.8 ± 0.1	0.81	0.88	0.79
	Corrected LV mass, g	1.0 ± 0.1	1.3 ± 0.05	1.5 ± 0.06	1.4 ± 0.09	1.4 ± 0.07	1.0 ± 0.1	1.2 ± 0.04	1.3 ± 0.06	1.2 ± 0.06	1.3 ± 0.07	**0.03**	**0.04**	0.17
Female Offspring	IVSd, mm	1.9 ± 0.1	1.9 ± 0.1	1.8 ± 0.0	1.9 ± 0.1	1.9 ± 0.1	1.9 ± 0.1	1.9 ± 0.1	1.8 ± 0.0	1.9 ± 0.1	1.9 ± 0.0	0.32	0.60	0.95
	IVSs, mm	3.2 ± 0.2	3.2 ± 0.1	3.4 ± 0.1	3.4 ± 0.1	3.3 ± 0.1	3.2 ± 0.2	3.2 ± 0.1	3.3 ± 0.1	3.2 ± 0.1	3.3 ± 0.1	0.69	0.49	0.99
	LVIDd, mm	6.1 ± 0.2	6.7 ± 0.2	6.8 ± 0.2	7.3 ± 0.2	7.4 ± 0.2	6.1 ± 0.2	6.2 ± 0.3	7.1 ± 0.1	7.1 ± 0.3	7.0 ± 0.2	**0.003**	0.46	0.25
	LVIDs, mm	2.8 ± 0.1	3.0 ± 0.2	2.8 ± 0.2	3.4 ± 0.2	3.3 ± 0.3	2.8 ± 0.1	2.8 ± 0.3	3.1 ± 0.1	3.6 ± 0.3	3.3 ± 0.3	**0.04**	0.76	0.47
	LVPWd, mm	2.3 ± 0.2	1.7 ± 0.1	2.1 ± 0.1	2.1 ± 0.2	1.9 ± 0.1	2.3 ± 0.2	2.0 ± 0.1	2.0 ± 0.1	1.9 ± 0.1	2.1 ± 0.1	0.37	0.57	0.11
	LVPWs, mm	3.8 ± 0.1	3.1 ± 0.1	3.5 ± 0.1	3.6 ± 0.2	3.6 ± 0.2	3.8 ± 0.1	3.2 ± 0.1	3.5 ± 0.1	3.2 ± 0.1	3.5 ± 0.2	0.08	0.50	0.33
	Corrected LV mass, g	0.72 ± 0.03	0.69 ± 0.03	0.77 ± 0.04	0.92 ± 0.06	0.82 ± 0.04	0.72 ± 0.03	0.72 ± 0.04	0.79 ± 0.04	0.77 ± 0.03	0.82 ± 0.05	**0.006**	0.74	**0.05**

Times (in months) reflect duration of BrSp feeding. Values are expressed as means ± SEM; *n* = 6–14 animals/group. Male or female offspring in control and BrSp supplemented groups were compared over time with a 2-way ANOVA. LV, left ventricular; IVSd and IVSs, interventricular septum at diastole and systole, respectively; LVIDd and LVIDs, LV internal diameter at diastole and systole, respectively; LVPWd and LVPWs, LV posterior wall at diastole and systole, respectively.

**Table 3:** Systolic and diastolic function in rats fed a control or BrSp diet.

		Ctl					BrSp					*p-*Value		
		0 Months	6 Months	12 Months	16 Months	18 Months	0 Months	6 Months	12 Months	16 Months	18 Months	Time	BrSp	Int
Male Offspring	LV end-diastolic volume, µL	340.6 ± 25.9	389.6 ± 24.5	427.5 ± 19.4	471.8 ± 29.5	404.1 ± 32.1	340.6 ± 25.9	380.1 ± 20.2	355.5 ± 14.6	369.9 ± 44.5	412.2 ± 41.8	0.51	0.14	0.11
	LV end-systolic volume, µL	63.2 ± 19.0	84.4 ± 15.1	96.6 ± 15.7	96.6 ± 13.6	76.4 ± 13.2	63.2 ± 19.0	66.2 ± 10.9	54.3 ± 4.9	69.8 ± 14.8	59.9 ± 10.6	0.79	0.12	0.50
	Hear rate (bpm)	394.3 ± 7.8	332.1 ± 14.6	333.6 ± 12.6	328.9 ± 5.4	325.8 ± 17.7	426.2 ± 0.7	351.1 ± 9.8	315.8 ± 12.1	288.2 ± 15.8	309.5 ± 17.7	0.06	0.30	0.14
	Stroke volume, mL	277.4 ± 10.2	305.2 ± 13.1	330.9 ± 14.8	375.2 ± 28.0	327.6 ± 21.6	277.4 ± 10.2	313.9 ± 13.0	301.2 ± 10.8	300.1 ± 33.7	328.4 ± 24.3	0.55	0.19	0.13
	Cardiac output, mL/min	113.6 ± 2.9	100.5 ± 4.5	110.4 ± 6.7	123.0 ± 8.5	106.0 ± 8.4	113.6 ± 2.9	109.7 ± 4.6	94.5 ± 3.9	84.1 ± 6.9 **	102.9 ± 8.4	0.92	0.08	**0.003**
	Cardiac output, mL/min.g	0.18 ± 0.00	0.13 ± 0.01	0.14 ± 0.01	0.15 ± 0.01	0.14 ± 0.01	0.19 ± 0.00	0.14 ± 0.01	0.12 ± 0.01	0.10 ± 0.00	0.15 ± 0.02	0.17	0.34	**0.004**
	Mitral Flow Doppler													
	E, mm/s	918.4 ± 57.1	936.4 ± 49.7	886.2 ± 28.4	892.4 ± 76.4	917.1 ± 79.4	918.4 ± 57.1	838.1 ± 34.7	861.5 ± 40.5	807.1 ± 67.9	912.1 ± 90.2	0.72	0.24	0.77
	A, mm/s	998.1 ± 50.1	747.4 ± 72.2	754.4 ± 69.7	758.2 ± 86.6	827.0 ± 81.7	964.4 ± 0.0	718.9 ± 39.6	665.3 ± 51.7	485.4 ± 34.5	714.5 ± 72.8	0.18	0.10	0.48
	E/A	0.93 ± 0.08	1.31 ± 0.08	1.25 ± 0.11	1.08 ± 0.06	1.06 ± 0.06	0.93 ± 0.08	1.18 ± 0.05	1.32 ± 0.08	1.57 ± 0.13	1.28 ± 0.11	0.46	0.12	**0.03**
	TEI index	0.52 ± 0.05	0.44 ± 0.03	0.57 ± 0.04	0.46 ± 0.02	0.52 ± 0.04	0.52 ± 0.05	0.46 ± 0.04	0.55 ± 0.02	0.51 ± 0.06	0.57 ± 0.06	**0.02**	0.41	0.83
Female Offspring	LV end-diastolic volume, µL	190.4 ± 9.2	240.9 ± 16.6	250.4 ± 12.8	289.8 ± 21.4	294.8 ± 14.7	194.0 ± 29.9	220.0 ± 17.5	258.8 ± 10.8	279.2 ± 20.8	274.4 ± 14.6	**<0.001**	0.61	0.55
	LV end-systolic volume, µL	20.0 ± 4.0	33.9 ± 4.9	29.2 ± 5.6	47.1 ± 6.6	40.2 ± 9.4	17.3 ± 4.8	28.4 ± 5.4	32.9 ± 3.8	55.8 ± 12.8	44.9 ± 9.3	**0.007**	0.62	0.62
	Hear rate (bpm)	505.7 ± 11.6	398.5 ± 12.9	403.3 ± 15.0	370.4 ± 21.1	370.3 ± 11.5	484.3 ± 10.8	383.6 ± 19.8	403.3 ± 15.9	357.2 ± 16.3	332.5 ± 26.7	**0.004**	0.29	0.76
	Stroke volume, mL	170.4 ± 5.2	207.0 ± 12.7	221.2 ± 8.7	242.6 ± 17.4	258.2 ± 13.8	176.7 ± 25.0	191.6 ± 15.1	225.9 ± 8.6	213.3 ± 12.3	229.5 ± 9.3	**0.002**	0.17	0.42
	Cardiac output, mL/min	86.2 ± 4.6	82.7 ± 6.0	88.5 ± 3.4	89.3 ± 7.0	95.9 ± 6.4	85.3 ± 10.2	72.4 ± 5.9	90.6 ± 4.4	76.2 ± 5.4	76.8 ± 7.2	0.09	**0.04**	0.24
	Cardiac output, mL/min.g	0.20 ± 0.02	0.17 ± 0.1	0.16 ± 0.01	0.16 ± 0.01	0.18 ± 0.02	0.21 ± 0.03	0.15 ± 0.01	0.17 ± 0.01	0.14 ± 0.01	0.14 ± 0.2	0.48	0.06	0.22
	Mitral Flow Doppler													
	E, mm/s	824.2 ± 0.0	837.1 ± 47.2	885.5 ± 56.3	827.1 ± 72.4	786.4 ± 43.9	832.6 ± 0.0	780.1 ± 44.6	889.0 ± 42.9	902.5 ± 53.7	797.9 ± 61.1	0.13	0.99	0.77
	A, mm/s	906.5 ± 0.0	854.4 ± 46.5	760.4 ± 60.2	661.4 ± 54.2	789.3 ± 41.2	926.7 ± 0.0	778.5 ± 51.9	764.0 ± 60.7	660.0 ± 42.8	766.9 ± 83.4	0.12	0.58	0.91
	E/A	0.91 ± 0.00	0.96 ± 0.03	1.24 ± 0.20	1.27 ± 0.13	1.05 ± 0.08	0.90 ± 0.00	1.10 ± 0.06	1.20 ± 0.10	1.46 ± 0.11	1.30 ± 0.20	0.10	0.28	0.75
	TEI index	0.54 ± 0.05	0.48 ± 0.05	0.58 ± 0.04	0.55 ± 0.07	0.54 ± 0.05	0.54 ± 0.05	0.44 ± 0.03	0.50 ± 0.03	0.50 ± 0.07	0.50 ± 0.03	0.11	0.22	0.96

Times (in months) reflect duration of BrSp feeding. Values are expressed as means ± SEM; *n* = 6–14 animals/group. Male or female offspring in control and BrSp supplemented groups were compared over time with a 2-way ANOVA.

**Table 4:** Behavioral analysis of rats subjected to open field, elevated plus maze, and tapered beam tests.

Behavioral Test	Parameters	Male			Female		
		Ctl	BrSp	*p*-Value	Ctl	BrSp	*p*-Value
Open Field Test	Total distance traveled (m)	13.6 ± 1.9	16.2 ± 2.9	0.44	20.1 ± 1.2	21.8 ± 1.4	0.35
	Time mobile (s)	53 ± 5	77 ± 10	**0.03**	79.11 ± 4.46	88 ± 4	0.13
	Time in inner zone (s)	37 ± 10	48 ± 12	0.49	27.69 ± 3.16	32 ± 5	0.48
Elevated Plus Maze	Time in open arm (s)	118 ± 29	92 ± 26	0.41	125 ± 20	150 ± 21	0.41
	Time in closed arm (s)	125 ± 20	150 ± 21	0.53	99 ± 19	100 ± 19	0.98
	Entries into open arm	6.4 ± 0.8	7.0 ± 1.1	0.66	9.4 ± 0.8	11.3 ± 1.6	0.35
	Entries into closed arm	5.8 ± 0.7	5.9 ± 1.5	0.97	8.4 ± 1.1	7.0 ± 0.8	0.30
	Open arm avoidance Index	53.9 ± 5.8	43.8 ± 8.2	0.31	52.1 ± 5.4	43.6 ± 5.7	0.30
Tapered beam	Total foot faults	3.4 ± 0.6	3.5 ± 0.5	0.86	1.3 ± 0.4	0. 9 ± 0.2	0.43
	Right foot faults	1.9 ± 0.4	2.1 ± 0.6	0.80	0.5 ± 0.2	0.3 ± 0.1	0.39
	Left foot faults	1. 5 ± 0.3	1.4 ± 0.4	0.97	1.0 ± 0.3	0.4 ± 0.1	0.06

Values are expressed as means ± SEM; *n* = 7–10 animals/group. Behavioral data for male and female rats in control and BrSp groups is analyzed by student’s *t*-test at t = 18 months of age.

## Data Availability

Data and information related to this study are available upon reasonable request from corresponding authors.

## Supplementary Materials

The following supporting information can be downloaded at: https://www.mdpi.com/article/10.3390/ijerph192013468/s1, Table S1: Criteria for the humane end point and euthanasia in rats. Table S2: Observed morbidities at the time of euthanasia in control and BrSp-fed rats.
