# Supplementary material for: Broccoli Sprouts Promote Sex-Dependent Cardiometabolic Health and Longevity in Long-Evans Rats
Source: Int J Environ Res Public Health. 2022 Oct 18;19(20):13468. doi: 10.3390/ijerph192013468 (PMC9603818; doi:10.3390/ijerph192013468)
Supplement: Supplementary file 1 [file ijerph-19-13468-s001.zip › ijerph-1952020-supplementary.pdf]

Supplementary materials to:

Broccoli Sprouts Promote Sex-Dependent Cardiometabolic Health and Longevity in Long-Evans Rats

Ronan M. N. Noble [1,2,†], Forough Jahandideh [2,3,†], Edward A. Armstrong [1], Stephane L. Bourque [1,2,3,4,*] and Jerome Y. Yager [1,2,*]

Table S1. Criteria used for the humane endpoint and euthanasia in rats

Parameter	Condition
Weight loss	Body weight loss of more than 20% from their top weight
Breathing abnormalities	Respiratory distress, labored breathing, increased or decreased respiratory rate, cyanosis
Bleeding/open sores	When there is a prolapse of rectum/vagina/penis or rat cannot urinate
Tumor volume or burden	Tumor(s) ulcerated, necrotic, exceeding 3 cm or impairing function (interfere with quality of life)
Limb paralysis	
Dermatitis	
Malocclusion	

Table S2. Observed morbidities at the time of euthanasia in control and BrSp-fed rats

Groups	ID	Age (days)	Humane endpoint	Cardiopulmonary	Kidney	Liver	Brain	Tumor	Other conditions
Male-Ctl	1	608	Weight Loss	1	1				
	2	570	Weight Loss	1	1				
	3	779	Breathing Problems/pain	1		1		1	Hemangiosarcoma
	4	779	Unknown (found dead)						
	5	752	Weight Loss		1				
	6	612	Weight Loss	1	1				
	7	617	Movement issues				1		
	8	672	Rear leg paralysis	1	1				
	9	706	Weight Loss	1	1		1	1	
	10	679	Tumor	1	1			1	
	11	540	Weight Loss	1	1		1		
	12	442	Weight loss	1	1				
	13	651	Weight Loss	1	1	1	1		Fast Clotting blood
	14	675	Weight Loss	1	1	1			
Sum				11	11	3	4	3	
Male-BrSp	1	501	Unknown (found dead)						
	2	559	Weight Loss/problems Breathing	1					
	3	452	Unknown (found dead)					1	
	4	809	Weight Loss	1				1	
	5	849	Weight Loss	1	1	1			
	6	884	Weight Loss	1	1		1		
	7	502	Unknown (found dead)						
	8	612	Weight Loss	1					
	9	812	Problems Breathing/not eating	1		1			
	10	801	Malocclusion	1	1	1		1	
	11	635	Weight Loss		1				
	12	545	Weight Loss	1	1				
	13	619	Weight Loss	1	1				
	14	675	Weight Loss	1	1	1			
Sum				10	7	4	1	3	
Female-Ctl	1	641	Malocclusion						
	2	557	Malocclusion						
	3	793	Tumor					1	
	4	840	Weight Loss	1	1		1		Porphyrin from nose
	5	855	Tumor	1		1		1	
	6	612	Tumor	1				1	
	7	690	Weight Loss					1	
	8	576	Tumor	1				1	
	9	899	Malocclusion	1			1		
	10	476	Difficulty breathing	1					

	11	741	Weight Loss/pain	1	1	1	1	1	
	12	716	Unknown (found dead)						
	13	892	Weight Loss	1	1				
	14	850	Weight Loss	1	1	1		1	
Sum				9	4	3	3	7	
Female-BrSp	1	826	Tumors	1				1	
	2	608	Malocclusion						
	3	638	Dermatitis	1					Dermatitis
	4	872	Weight Loss		1	1	1		
	5	856	Tumor					1	
	6	884	Impaired hindlimb movement		1		1		Loss of hindlimb function
	7	945	Impaired hindlimb movement						
	8	956	Unknown (found dead)						
	9	619	Tumor					1	
	10	866	Tumor	1	1			1	
	11	803	Weight Loss	1	1		1		Abnormal stomach
	12	721	Tumors		1			1	
	13	903	Malocclusion		1				
	14	850	Tumor	1	1			1	
Sum				5	7	1	3	6	
